# Association between the Use of Dipeptidyl Peptidase 4 Inhibitors and the Risk of Dementia among Patients with Type 2 Diabetes in Taiwan

**DOI:** 10.3390/jcm9030660

**Published:** 2020-02-29

**Authors:** Kuan-Chan Chen, Chi-Hsiang Chung, Chieh-Hua Lu, Nian-Sheng Tzeng, Chien-Hsing Lee, Sheng-Chiang Su, Feng-Chih Kuo, Jhih-Syuan Liu, Chang-Hsun Hsieh, Wu-Chien Chien

**Affiliations:** 1 Division of Endocrinology and Metabolism, Department of Internal Medicine, Tri-Service General Hospital, National Defense Medical Center, Taipei 11490, Taiwan; kuanchanndmc@gmail.com (K.-C.C.); undeca2001@gmail.com (C.-H.L.); doc10383@gmail.com (C.-H.L.); doc10504@gmail.com (S.-C.S.); shoummie@hotmail.com (F.-C.K.); ajleonn21@hotmail.com.tw (J.-S.L.); 2 School of Public Health, National Defense Medical Center, Taipei 11490, Taiwan; g694810042@gmail.com; 3 Department of Psychiatry, Tri-Service General Hospital, School of Medicine, National Defense Medical Center, Taipei 114, Taiwan; pierrens@mail.ndmctsgh.edu.tw; 4 Student Counseling Center, National Defense Medical Center, Taipei 11490, Taiwan; 5 Department of Medical Research, Tri-Service General Hospital, National Defense Medical Center, Taipei 114, Taiwan; 6 Graduate Institute of Life Science, National Defense Medical Center 11490, Taipei, Taiwan

**Keywords:** alzheimer’s disease, diabetes mellitus, DPP-4 inhibitor, dementia

## Abstract

Study Objectives: Diabetes mellitus per se and its related therapy have been frequently associated with an increased risk of developing dementia. However, studies that explored the risk of dementia from the use of the novel oral antidiabetic medication dipeptidyl peptidase 4 inhibitor (DPP-4i) have been limited, especially in Asian populations. The present study aimed to determine the effect of DPP-4i on the subsequent risk of dementia among patients with type 2 diabetes (T2D) in Taiwan. Methods: This study utilized data from the Longitudinal Health Insurance Database between 2008 and 2015. We enrolled 2903 patients aged ≥50 years, who were on DPP-4i for a diagnosis of T2D and had no dementia. A total of 11,612 subjects were included and compared with a propensity score-matched control group who did not use DPP-4i (non-DPP-4i group). Survival analysis was performed to estimate and compare the risk of dementia—including Alzheimer’s disease, vascular dementia, and other dementia types—between the two groups. **Results:** Both groups had a mean age of 68 years, had a preponderance of women (61.8%), and were followed up for a mean duration of 7 years. The risk of all-cause dementia was significantly lower in the DPP-4i group than in the non-DPP-4i group (hazard ratio (HR) 0.798; 95% confidence interval (CI) 0.681–0.883; p < 0.001), with a class effect. This trend was particularly observed for vascular dementia (HR 0.575; 95% CI 0.404–0.681; p < 0.001), but not in Alzheimer’s disease (HR 0.891; 95% CI 0.712–1.265; p = 0.297). The Kaplan–Meier analysis showed that the preventive effect on dementia was positively correlated with the cumulative dose of DPP-4i. **Conclusions:** DPP-4i decreased the risk of dementia with a class effect, especially vascular dementia, but not in Alzheimer’s disease. Our results provide important information on the drug choice when managing patients with T2D in clinical practice.

## 1. Introduction

Diabetes mellitus is one of the major global health problems in an aging society. Driven by aging and aging-related comorbidities, diabetes mellitus continues to increase in prevalence, not only in Taiwan, but also around the world. In Taiwan, the prevalence of type 2 diabetes (T2D) was reported by community studies to be up to 26.9% among those aged over 65 years [1]. In addition, the prevalence of dementia accounted for 2% to 5% of the population aged over 65 years [2], and this has placed a heavy burden on dementia patients, their caregivers and the community [3,4,5].

Alzheimer’s disease (AD) and vascular dementia are the most common types of dementia and share both vascular and neurodegenerative pathophysiological mechanisms [6,7]. Excluding the vascular mechanism, the other postulated mechanisms for the association between AD and T2D include hyperglycemia, inflammation, brain insulin resistance, the reduced degradation of β-amyloid peptides secondary to the formation of advanced glycation end products, and competition for the insulin-degrading enzyme; in fact, AD is being considered as type 3 diabetes [8,9]. A previous study disclosed that diabetes mellitus was associated with a 1.7-fold greater risk of dementia among elderly people [10]. Furthermore, T2D was a significant risk factor for not only AD, but also for vascular dementia [11].

In both nondiabetic and T2D subjects, there are several risk factors for dementia development [12]. T2D-related therapy and its comorbidities were associated with an increasing incidence of dementia [13]. There were a few studies that explored the risk factors and/or validated a practical risk score to predict the future risk of dementia among individuals with T2D [14]. Among these risk factors, antidiabetic medications were extensively evaluated for their potential risks and benefits. However, the results remained controversial for old antidiabetic medications. For example, there were studies that demonstrated a diminished risk of dementia with metformin use [15], but these benefits were found in the age group of <75 years and in patients on high-dose metformin [16,17]. These results for other antidiabetic medications were also inconclusive, including for sulfonylureas and thiazolidinediones [8,18,19].

On the other hand, studies that have explored the role of dipeptidyl peptidase 4 inhibitors (DPP-4i) on dementia development in patients with T2D have been limited. A previous animal study reported that DPP-4i improved the functions of the neuronal insulin receptor and the brain mitochondria, and that it can delay some forms of AD-like pathology [20]. In addition, sitagliptin was reported to have a possible association with the improvement of cognitive function in elderly diabetic patients with and without AD [21]. However, a recent study revealed that therapy with linagliptin did not improve the cognitive performance of patients with T2D and cardiorenal disease [22]. According to present evidence, the effects of DPP-4i on dementia remain inconclusive. In addition, it remains to be clarified whether the association of DPP-4i with dementia is a class effect or is affected by other factors, such as treatment duration.

Therefore, we carried out a study to investigate the association between DPP-4i use and the different types of dementia, as well as the impact of the different types and therapeutic durations of DPP-4i among patients with diabetes. We assessed the incidence of dementia in diabetic patients aged ≥50 years and on DPP-4i use, on the basis of the National Health Insurance Research Database (NHIRD), which was a claims database that was retrieved from the entire population of Taiwan.

## 2. Materials and Methods

### 2.1. Data Sources

In this study, we used 7 year data from the NHIRD, based on the two million included in the Longitudinal Health Insurance Database (LHID) in Taiwan (2008 to 2015). The National Health Insurance (NHI) Program was launched in Taiwan in 1995, and as of June 2009, included contracts with 97% of the medical providers, with approximately 23 million beneficiaries, or more than 99% of the entire population [23]. The NHIRD uses the International Classification of Diseases, Ninth Revision, Clinical Modification (ICD-9-CM) codes to record the diagnoses. All diagnoses of T2D were made by board-certified internists, endocrinologists, or other related specialists, and all diagnoses of dementia were made by board-certified psychiatrists or neurologists. The NHI administration randomly reviews the records of ambulatory care visits and inpatient claims periodically to verify the accuracy of the diagnoses. The NHIRD was therefore considered appropriate for this study on the association between dementia and use of DPP-4i.

### 2.2. Study Design and Sampled Participants

Sitagliptin was the first DDP-4i approved by the United States Food and Drug Administration on October 16, 2006, and has been marketed in Taiwan since 2008. This study was a retrospective matched-cohort design between January 1, 2008 and December 31, 2015. From the LHID, we identified and selected 5028 patients with diabetes (ICD-9-CM 250.XX) and who used DPP-4i for >90 days. Each enrolled patient was required to have made at least three outpatient visits within the study period. The diabetic patients who were <50 years old, those previously diagnosed with dementia, and those who were on DPP-4i before the index date were excluded. In addition, patients who received GLP-1 agonist before the tracking were excluded.

**Figure 1:**
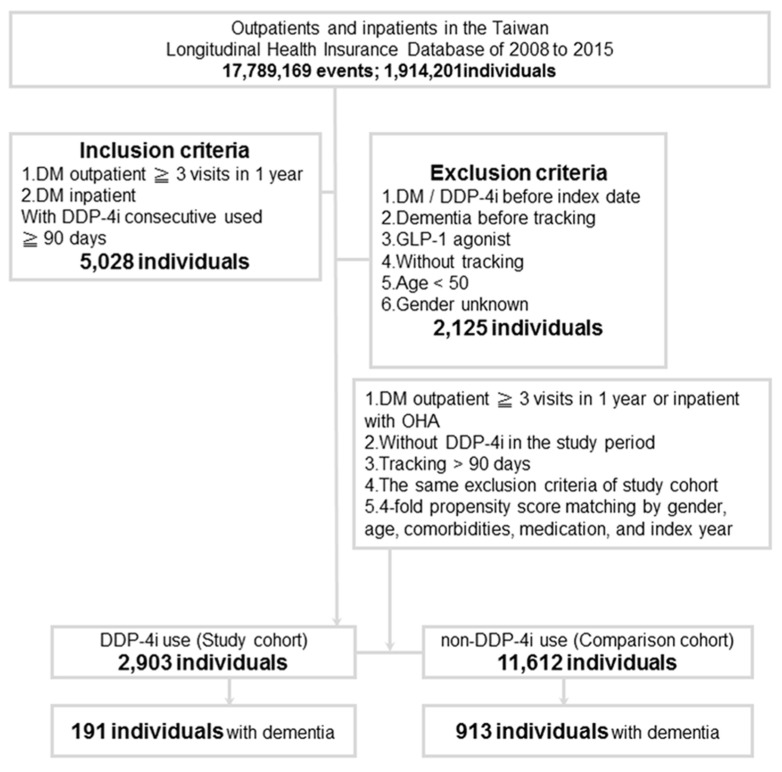
A flow chart of the sample selection process. DM = diabetes mellitus; DPP-4i = dipeptidyl peptidase 4 inhibitor; GLP-1 agonist = glucagon-like peptide 1 agonist; OHA = Oral Hypoglycemic Agents.

Data on the use of DPP-4i, including sitagliptin, linagliptin, saxagliptin, vildagliptin, and alogliptin, were collected. This study enrolled 2903 patients on DPP-4i use (DPP-4i group), and 11,612 patients without DPP-4i use (non-DPP-4i group) were matched by 1:4 propensity score matching, using confounding variables including sex, age, comorbidities, medication and index year. (Figure 1).

### 2.3. Comorbidity

The comorbidities included hypertension, hyperlipidemia, obesity, microvascular or macrovascular complications of diabetes, heart failure, depression, hearing loss, and atrial fibrillation. The microvascular complications included retinopathy, nephropathy, and neuropathy, whereas the macrovascular complications included stroke, coronary artery disease, and peripheral atrial occlusive disease. In addition, the Charlson comorbidity index was used to categorize the comorbidities based on the ICD-9-CM codes; the scores for each comorbidity category were combined into a single comorbidity score. A score of zero indicated that no comorbidities were found, and higher scores indicated a higher comorbidity burden [24].

### 2.4. Outcome Measurements

All study participants were followed up from the index date until the diagnosis of dementia (ICD 9-CM codes: 290.0, 290.10, 290.11, 290.12, 290.13, 290.20, 290.21, 290.3, 290.41, 290.42, 290.43, 290.8, 290.9, and 331.0), withdrawal from the NHI program, or at the end of 2015. These patients with dementia were grouped as AD (ICD-9-CM331.0), vascular dementia (ICD-9-CM 290.41–290.43), and other dementia (ICD-9-CM 290.0, 290.10–290.13, 290.20–290.21, 290.3, 290.8–290.9).

### 2.5. Statistical Analysis

All analyses were performed using the SPSS software version 22 (SPSS Inc., Chicago, IL, USA). The χ^2^ test and Student’s t-test were used to evaluate the distribution of the categorical and continuous variables, respectively. The Fisher’s exact test was used to analyze the differences in categorical variables between the two cohorts. Multivariate Cox proportional hazards regression analysis was used to determine the risk of dementia, and the results were presented as hazard ratios (HR) with 95% confidence intervals (CI). The difference in the risk of dementia between the DPP-4i and the non-DPP-4i groups was estimated using the Kaplan–Meier method with the log-rank test. A two-tailed *p* value of <0.05 was considered to indicate statistical significance.

### 2.6. Ethics Approval

This study was conducted in accordance with the Code of Ethics of the World Medical Association (Declaration of Helsinki). The institutional review board of the Tri-Service General Hospital approved this study (IRB No. 2-107-05-026 and IRB No. 1-106-05-169).

## 3. Results

**Figure 2:**
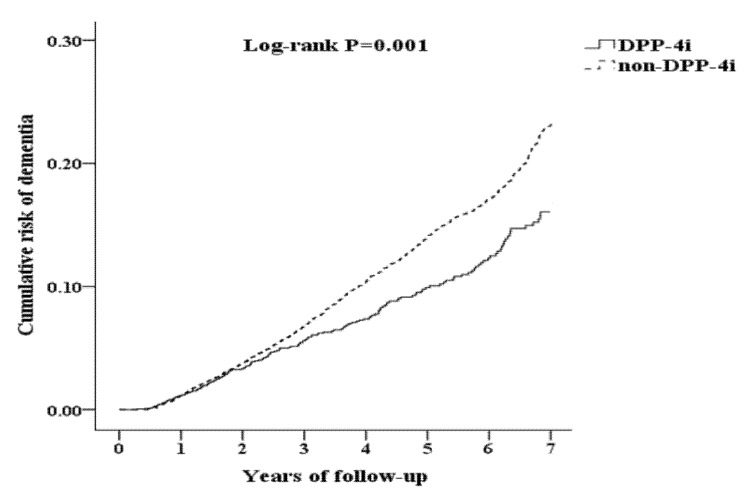
Kaplan–Meier curves for the cumulative risk of dementia in diabetic patients with or without dipeptidyl peptidase 4 inhibitor use.

Table 1 demonstrates the demographics of the study population. There were 2903 patients in the DPP-4i group and 63,430 patients in the non-DPP-4i group. After propensity score matching at a ratio of 1:4, 11,612 pairs remained. Both groups had a mean age of 68 years, had female preponderance (61.8%), and were followed up for a mean duration of 7 years. Of the 14,515 enrollees, dementia developed in 191 (6.5%) of the study subjects and in 913 (7.8%) of the matched controls. Figure 2 demonstrates that the cumulative incidence of dementia was lower in the DPP-4i group than in the non-DPP-4i group (*p* < 0.001).

Table 2 shows the Cox regression analysis of the association between DPP-4i use and the risk of developing different kinds of dementia. The crude HR of overall dementia was 0.772 (95% CI 0.663–0.856; *p* < 0.001) and remained significant after adjusting for confounding variables (adjusted HR 0.798; 95% CI 0.681–0.883; *p* < 0.001). Compared with the non-DPP4i group, the DPP-4i group had associated decreased risks for developing vascular dementia (adjusted HR 0.575; 95% CI 0.404–0.681; *p* < 0.001) and the other types of dementia (adjusted HR 0.684; 95% CI 0.556–0.749; *p* < 0.001), but not AD (adjusted HR 0.891; 95% CI 0.712–1.265; *p* = 0.297). The results were the same, even after the exclusion of individuals who were diagnosed as having dementia within the first year and the first 5 years. The reduction in the risk of dementia development in the DPP-4i group was independent of age, sex, and the presence of microvascular and macrovascular complications (Table 3).

Table 4 shows the comparison of the risk of dementia among the different types of DPP-4i. All types of DPP-4i had significant protective effects against dementia development. Moreover, there was a cumulative protective effect of all of the types of DPP-4i, especially when the duration of therapy was greater than 1 year. The associated decreased risk of dementia with DPP-4i use for less than 1 year was observed only in patients treated with sitagliptin or saxagliptin.

## 4. Discussion

Our study demonstrated that compared with non-DPP-4i use, DPP-4i use was associated with a 21% lower risk of developing all types of dementia in older patients with T2D. Furthermore, the DPP-4i types seemed to have a class effect that was dose-dependent. This information may be important for physicians when choosing the optimal antidiabetic medications for patients of T2D with or without dementia. 

DPP-4 inhibitor, which is one of the incretin-based therapies that increase the concentration of circulating GLP-1, has been frequently used for the treatment of T2D because of its relatively low risk of inducing hypoglycemia [25]. Our results demonstrated that the types of DPP-4i had a class effect on dementia prevention, with a main focus on vascular dementia, but not on AD. These results were not consistent with those of previous human studies [21,22,26]. The discrepancy among the studies may be attributed to the populations and numbers of the study participants, study designs, durations of follow up, and end points of the studies (cognition and dementia definitions and types). The CARMELINA-COG study is the only randomized controlled trial to evaluate the effect of using DPP-4i on cognition function, and the results showed that linagliptin therapy did not modulate cognitive decline in patients with T2D and cardiorenal comorbidities. This study used the Mini-Mental State Examination (MMSE) to evaluate cognitive function, instead of the risk of dementia development, with a mean 2.5 year follow-up [22]. Similarly, Isik et al. demonstrated that sitagliptin therapy improved the cognitive function of elderly patients with T2D, regardless of a prior history of AD. However, the duration of follow up was only 6 months [21]. The latest nested case control study from a cohort of 176,250 patients registered in the Danish National Diabetes Register study showed that the use of DPP-4i in T2D patients significantly decreased the risk of dementia compared to in non-DPP-4i users during a long duration of follow up (7.2 years). However, this study did not reveal the effect of DPP-4i on different types of dementia [26].

GLP 1 receptors are expressed in the neurons of the central nervous system. Stimulation of the GLP-1 receptor could attenuate neuroinflammation and enhance neurogenesis in diabetes-induced dementia, in animal models [27,28]. The concept was supported by a small randomized, controlled study in humans, which showed that liraglutide therapy improved memory function in obese patients with prediabetes or early type 2 diabetes, independently of weight loss and the glycemic lowering effect [29]. Therefore, increased circulating GLP 1 concentrations from DPP-4i therapy may have a neuroprotective role in the treatment of dementia. According to previous animal experimental studies, the neuroprotective effects of DPP-4i were evident in improving learning behavior and memory in AD and/or vascular dementia among the different types of DPP-4i [30,31,32,33,34,35,36]. The mechanisms of DPP-4i on vascular dementia found in animal studies included attenuating endothelial dysfunction, reducing cerebral oxidative stress, and decreasing ischemic brain damage, via a glucose-independent mechanism, likely involving GLP-1 [30,33,34]. Moreover, the complex pathophysiology of AD, which involves vascular and non-vascular mechanisms, is documented and characterized by the accumulation of aggregated amyloid-β (Aβ) protein and hyper-phosphorylated tau protein, neuroinflammation, and a reduction in cerebral glucose consumption [37]. DPP4 might play (a) role(s) in amyloid formation according to an in vitro experiment, and DPP-4i decreased amyloid-β deposition and tau phosphorylation by increasing hippocampal GLP-1 levels [36]. These results implied the beneficial effects of DPP-4i therapy on AD development. Furthermore, not all of the previous animal studies showed neuroprotection with DPP-4i. A previous animal study showed that sitagliptin may increase the risk of dementia by aggravating tau phosphorylation and insulin resistance in the brain [38]. Therefore, the complex pathophysiological mechanism involved in AD development may partly account for the absence of a diminished risk of AD from DPP 4i therapy in the current study.

Thus, although it is generally considered that the use of DPP 4i has a beneficial impact on cognition function, it still remains inconclusive that there is a beneficial effect of DPP 4i therapy on diminishing the risk of different types of dementia development, according to the current experimental and clinical studies. A further, large-scaled, RCT trial should be designed to clarify this issue.

Hypoglycemia is considered to be an independent risk factor for dementia development [39]; the risk of dementia in DPP-4i users should be lower than in users of other, older oral antidiabetic agents. A Korean cohort study showed that compared with sulfonylurea, DPP-4i decreased the risk of dementia in elderly patients with T2D. However, contrary to this present study, that study showed benefits in AD but not in vascular dementia [40]. The results are similar to those from another retrospective longitudinal study of metformin-based therapy, in which adding DPP-4i was shown to better protect against worsening cognitive function than sulfonylureas, in older patients with T2D and mild cognitive impairment [41]. Sulfonylureas have been associated with the risk of hypoglycemia, which had been commonly overused in older patients [42]. In addition, glycemic fluctuations might contribute to poor cognitive outcomes [43]. On the other hand, compared with other oral antidiabetic agents, especially sulfonylureas, DPP-4i provides steady glucose variability [44,45]. Accordingly, our study results demonstrate the independent benefits of DPP-4i use in preserving the cognitive function of patients with diabetes.

In our subgroup analysis, the association between DPP-4i use and the decreased risk of dementia was evident in patients aged ≥50 years, regardless of the presence of diabetes complications. Nevertheless, we found that the benefits of DPP-4i were weaker in those ≥65 years old and in those with macrovascular or microvascular complications of diabetes. The results were similar to those in a recent study that showed that overall, linagliptin did not reduce cognitive decline in patients with T2D and cardiorenal comorbidity [22]. Therefore, the protective effect of DPP-4i against dementia may be greater in younger patients and those without diabetic complications. 

There were several limitations of this study. Although these insurance claims data allowed the identification of patients with dementia or diabetes, data on diabetes severity, duration, and impact of control, such as glycated hemoglobin levels, were not available. Owing to the retrospective design, patients with mild cognitive decline may have been underdiagnosed. Other residual confounding factors, such as education, genetics or dietary factors, and body mass index, were not included in the NHIRD. In addition, because this was a population-based study, the actual mechanism for the association between DPP-4i use and dementia in patients with T2D cannot be clarified.

In conclusion, compared with non-DPP-4i use, DPP-4i use was associated with a lower risk of dementia in older Taiwanese patients with T2D. Further study on the neuroprotective effects of DPP-4i, based on a longer follow-up period, may be necessary to validate the risk of dementia with DPP-4i use.

## Figures and Tables

**Table 1:** Baseline characteristics of the matched pairs.

	With DPP-4i use	Without DPP-4i use	*p*
N	2,903	11,612	
Number of men (percentage)	1,109 (38.20%)	4,436 (38.20%)	0.999
Age (years)	68.05 ± 8.75	68.01 ± 8.69	0.808
Age group (years)			0.999
50–64	1,247 (42.96%)	4,988 (42.96%)	
≥65	1,656 (57.04%)	6,624 (57.04%)	
Hypertension	28.28	28.12	0.861
Hyperlipidemia	11.85	12.03	0.789
Obesity	0.07	0.07	0.999
Macrovascular complications of DM	20.43	20.76	0.690
Microvascular complications of DM	22.94	22.50	0.613
Heart failure	2.00	2.02	0.953
Chronic kidney disease	17.43	17.28	0.852
Depression	4.06	3.91	0.701
Hearing loss	4.10	3.95	0.718
Atrial fibrillation	1.24	1.63	0.131
Obstructive sleep apnea	4.27	4.31	0.919
CCI_R	1.24 ± 1.74	1.26 ± 1.80	0.558
Medication used			
Metformin	98.00	98.16	0.582
Sulfonylureas	89.46	88.52	0.153
Glinide	78.50	77.39	0.199
Thiazolidinedione	54.53	53.21	0.203
AGi	19.70	19.98	0.740
SGLT-2i	18.98	18.32	0.410
Insulin	73.03	73.17	0.881

Categorical variables were compared using Chi-square or Fisher’s exact test. Continuous variables were compared using Student’s t-test. DM = diabetes mellitus; CCI_R = Charlson comorbidity index without dementia; DPP-4i = dipeptidyl peptidase 4 inhibitor; AGi=α-glucosidase inhibitor; SGLT-2i=Sodium–glucose co-transporter 2 inhibitor.

**Table 2:** The risk for dementia according to the use of DPP-4i.

	All Dementia Cases		Dementia in the First Year Excluded		Dementia in the First 5 Years Excluded	
Dementia Subgroup	Adjusted HR (CI)	*p*	Adjusted HR (CI)	*p*	Adjusted HR (CI)	*p*
**Overall dementia**	0.798 (0.681–0.883)	<0.001	0.811 (0.682–0.901)	<0.001	0.849 (0.700–0.927)	0.005
Alzheimer’s disease	0.891 (0.712–1.265)	0.297	0.986 (0.789–1.303)	0.297	1.265 (0.886–1.986)	0.596
Vascular dementia	0.575 (0.404–0.681)	<0.001	0.612 (0.423–0.705)	<0.001	0.631 (0.497–0.786)	<0.001
Other dementia	0.684 (0.556–0.749)	<0.001	0.756 (0.589–0.834)	<0.001	0.793 (0.643–0.912)	0.001

HR was adjusted according to the variables listed in the baseline. DPP-4i = dipeptidyl peptidase 4 inhibitor; HR = hazard ratio, CI = confidence interval.

**Table 3:** Subgroup analyses on the use of DPP-4i according to sex, age, and microvascular and macrovascular complications of DM.

DPP-4i	With		Without		With *vs.* without	
Group	Events	Rate	Events	Rate	Adjusted HR	*p*
**Total**	191	10.42	913	13.74	0.798 (0.681–0.883)	<0.001
**Sex**						
Male	66	10.14	326	14.11	0.756 (0.643–0.836)	<0.001
Female	125	10.58	587	13.54	0.822 (0.701–0.909)	<0.001
**Age group (years)**						
50–64	18	8.71	96	12.38	0.742 (0.601–0.801)	<0.001
≥65	173	10.64	817	13.92	0.895 (0.727–0.976)	0.026
**Macrovascular complications of DM**						
Without	124	9.43	575	12.36	0.713 (0.512–0.808)	<0.001
With	67	12.97	338	16.97	0.804 (0.695–0.937)	0.005
**Microvascular complications of DM**						
Without	176	12.72	861	16.13	0.706 (0.511–0.806)	<0.001
With	15	3.34	52	3.98	0.835 (0.718–0.962)	0.013

The rate was calculated per 1000 person-years. HR was adjusted according to the variables listed in the baseline characteristics DM = diabetes mellitus; DPP-4i = dipeptidyl peptidase 4 inhibitor; HR = hazard ratio; CI = confidence interval.

**Table 4:** Comparison of the risk for dementia according to the use of DDP-4i.

	Adjusted HR	Lower CI	Higher CI	*p*
**All DDP4i**	0.798	0.681	0.883	<0.001
**All DDP4i (90–364 days)**	0.874	0.746	0.967	0.027
Sitagliptin	0.749	0.672	0.928	0.013
Linagliptin	0.861	0.768	1.002	0.051
Saxagliptin	0.769	0.683	0.927	0.009
Vildagliptin	0.901	0.801	1.397	0.244
Alogliptin	0.984	0.870	1.495	0.395
**All DDP4i (≧365 days)**	0.780	0.665	0.864	<0.001
Sitagliptin	0.669	0.599	0.788	<0.001
Linagliptin	0.772	0.685	0.894	<0.001
Saxagliptin	0.687	0.610	0.828	<0.001
Vildagliptin	0.805	0.712	0.915	0.006
Alogliptin	0.879	0.778	0.971	0.031

HR was adjusted according to the variables listed in the baseline characteristics. DDP-4i = dipeptidyl peptidase 4; HR = hazard ratio; CI = 95% confidence interval.
